# Problems and challenges of predatory journals

**DOI:** 10.1111/jdv.15039

**Published:** 2018-05-29

**Authors:** G. Richtig, M. Berger, B. Lange‐Asschenfeldt, W. Aberer, E. Richtig

**Affiliations:** ^1^ Pharmacology Section Otto Loewi Research Center Medical University of Graz Graz Austria; ^2^ Department of Dermatology Medical University of Graz Graz Austria; ^3^ Department of Dermatology and Venereology State Hospital Klagenfurt Klagenfurt am Wörthersee Austria; ^4^ Department of Dermatology and Allergy Charité‐Universitaetsmedizin Berlin Berlin Germany

## Abstract

The companies publishing predatory journals are an emerging problem in the area of scientific literature as they only seek to drain money from authors without providing any customer service for the authors or their readership. These predatory journals try to attract new submissions by aggressive email advertising and high acceptance rates. But in turn, they do not provide proper peer review, and therefore, the scientific quality of submitted articles is questionable. This is important because more and more people, including patients, are reading such journals and rely on the information they provide. Consequently, predatory journals are a serious threat to the integrity of medical science, and it is crucial for scientists, physicians and even patients to be aware of this problem. In this review, we briefly summarize the history of the open access movement, as well as the rise of and roles played by predatory journals. In conclusion, young and inexperienced authors publishing in a predatory journal must be aware of the damage of their reputation, of inadequate peer review processes and that unprofitable journals might get closed and all published articles in that journal might be lost.

## Introduction

Since the 1950s, a continuous increase in the number of scientific manuscripts published every year has been observed.^1^ This may be attributed to rapid developments in scientific fields overall, and especially the medical field, but also to the fact that funding bodies rate scientists based on their publications. The latter rating is based on several parameters, such as the h‐Index, altmetric score, impact factor and citations. In particular, young researchers are placed under constant pressure to publish their work to increase their rating and receive funding.^2^, ^3^ Within this framework, an increasing number of companies that publish journals are aggressively trying to attract authors and encourage them to submit their valuable work to their scholarly, open access, peer reviewed journals. However, as the majority of these journals do not conduct proper peer review processes or offer customer service, these journals have been named ‘predatory journals’ by Jeffrey Beall – a librarian at the University of Colorado. He created a list of criteria, which could help authors recognize predatory journals and publishers.

Although these criteria are not exhaustive, in this review, we will summarize the results of research that has been conducted on predatory journals and provide an outlook on their potential to be recognized in the field of investigative journalism.

## The open access movement

Publishing practices have changed dramatically over recent years. Large, mainstream publishers of subscription‐based journals began to initially publish articles as online versions and then later as the respective print issues.^4^, ^5^ This was a reaction to the emergence of the open access (OA) movement in the 2000s.^4^ The OA model is characterized by the fact that journals make their articles widely available by distributing them in freely available forms online. The period of delay from submission to publication therefore is normally shorter compared to that of traditional journals; this guarantees faster dissemination and broader visibility of scientific work.^6^ However, in contrast to subscription‐based journals, which levy minor charges upon acceptance of the article, authors who submit their manuscripts to OA journals are required to pay a substantial publication fee, which is known as the article processing charge (APC).

There are several different APC models (detailed in Table 1): Firstly, the ‘gold’ OA model requires the author to pay up to several thousand euros to retain the article copyright, assure that the article is completely and freely available online and can be distributed to everyone. Secondly, the ‘green’ OA model restricts the author's right of distribution, whereby he or she may only distribute their articles through their website(s) or third‐party repository sites. Several other models have been established including hybrid access, whereby the authors can pay for OA in a subscription‐based journal.^7^ Many journals now offer this option as it has been suggested that OA articles are more likely to be cited.^8^ However, there are also subscription‐based journals that make their articles freely available after a certain period of time (e.g. 1 year after publication).^9^ The increase in the number of OA journals has also been facilitated by several funding bodies including the National Institutes of Health (NIH), which has required authors to make their work publicly available via PubMed Central (PMC) in order to receive funding (inclusion criteria for PMC can be found in Table S1).^8^ One of the best‐known OA journals is *PLOS one*, which publishes around 30 000 articles each year. Other leading prestigious publishers, including the Nature Publishing Group or Elsevier, now have OA journals within their portfolio (including scientific reports, nature communications and others).

**Table 1:** Different open access publishing models^55^

**Gold open access** 56
The so‐called ‘gold open access’ was the initial form of OA, whereby the author(s) or authors institution pay an APC to the OA journal at the time of manuscript acceptance. Therefore, these fees are (ostensibly) used to cover the peer review and publication costs, while no revenues are generated by subscriptions. The publishing practices are similar to those used by subscription‐based publishers, although the peer review and publishing processes can be shorter (with no decline in quality)
**Green open access** 6, 56
In the ‘green open access’ model, authors who publish in a subscription journal are allowed to make a manuscript version of their article freely available on their website or an institutional repository site. Most journals already offer this model, as some research funders like the National Institutes of Health often insist on this option
**Hybrid model** 24
In this model, traditional, subscription‐based journals offer authors the possibility to make their articles openly accessible in the journal's electronic archive upon payment of an APC. Therefore, a subscription‐based journal may offer members without subscriptions free access to articles. This model was supposed to represent an intermediate solution between subscription‐based and open access journals

APC, article processing charge; OA, open access.

However, as the success of the OA journals mounted, several changes and problems arose. Firstly, traditional, subscription‐based journals have attracted members of their readership by delivering research articles of high quality and/or which are extremely interesting. As the primary source of revenue for the companies publishing these journals is institution or individual subscriptions, these journals have to deliver research articles of high quality. In the OA journals, the publisher subscription has been replaced by an APC paid by authors to get their work published. Because the APC received authors upon manuscript acceptance, represents the primary revenue of OA journals, editors of these journals are put under pressure to ensure timely peer review processes and fast‐track publication times. They need to contact as many authors as possible and encourage them to submit their articles to the journal. Secondly, OA journals have significantly lower production costs as the majority of OA journals are online‐only journals. By not providing any hard copies, costs may be minimized substantially through the evasion of printing expense, warehouse storage, distribution and shipping.^6^ However, they must obtain a specific number of successful submissions per month to cover these running costs. This situation inevitably increases the risk that OA journals rank scientific standards, including rigorous peer review processes, below the publication acceptance rate.^9^

## Predatory journals and Beall's list

**Figure 1:**
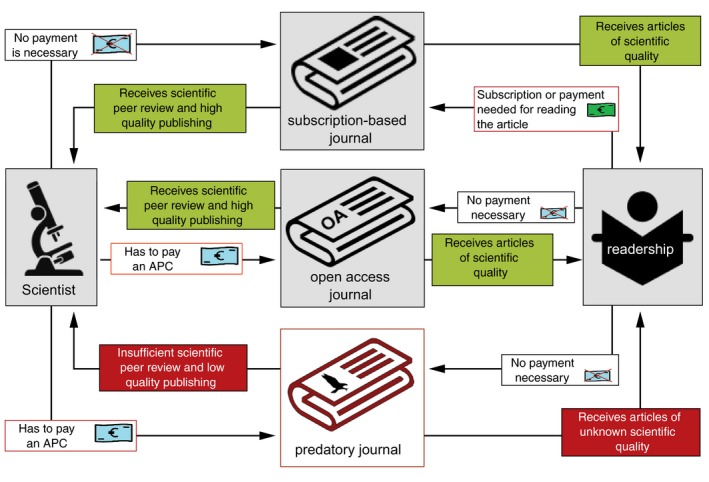
The relationship between researcher, journal and readership. Subscription‐based journal: Scientist submits his or her work to the journal without costs, and the journal provides high‐quality peer review to the authors to ensure the scientific quality of the submitted paper. Members of the readership receive peer review articles of high scientific quality, but have to pay a fee to access the journal's content. Open access journal: Same standards as a subscription‐based journal, but the author has to pay an article processing charge (APC) in this model, and the content is freely available to the readership in return. Predatory journal: Copy the open access publishing model by levying APCs on authors, but do not deliver high‐quality, peer reviewed articles (and other services) and do not ensure the scientific quality of submitted articles. Therefore, they are fooling the scientific system as well as members of the readership.

With the rise of open access and the movement to publish articles only online, an increasing number of publishers and journals that exploited the OA model emerged (see Fig. 1). Therefore, claims have been made that the peer review process may not be properly followed, because the companies producing these predatory journals are pursuing the major goal of obtaining a financial profit in the form of APC fees from the authors.^10^, ^11^

One illustrative example has been made by John Bohannon, who simultaneously submitted a mundane scientific article to 304 different OA journals. According to Bohannon, the article had so many ‘… grave errors that a competent peer reviewer should easily identify it as flawed and unpublishable’.^12^ But more than 50% of the journals accepted the article. The work of Bohannon highlighted several troubling issues. Firstly, in the majority of cases, only superficial peer review process was performed. This led to the fact that, secondly, the article was sold as ‘scientific’. Thirdly, journals belonging to well‐known publishers, such as Elsevier, Sage and Wolters Kluwer, also accepted the bogus article. Fourthly, it became obvious that the journal titles did not necessarily reflect the origin of the journal.

**Figure 2:**
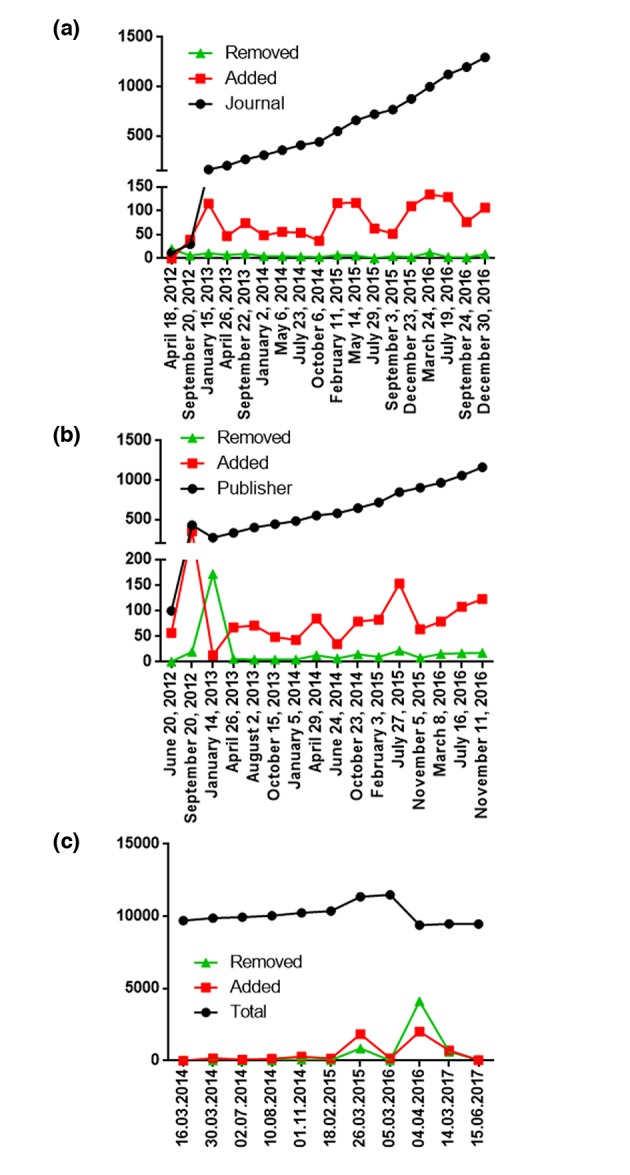
Overview of the total count of (a) journals and (b) publishers on Beall's list regarding predatory journals/publishers, and the number of titles added and removed over the last few years by Beall. (c) Total count of journals listed in the Directory of Open Access Journals, including additions and removals, over the last years. Cached data on Beall's original list (a and b) (https://scholarlyoa.com) and for the Directory of Open Access Journals (c) (https://doaj.org/) were obtained from an Internet archive: Wayback Machine (https://archive.org/web/), stored in Excel worksheets (Microsoft, Redmond, WA), analysed with a self‐written Python 2.7 (Python Software Foundations, Beaverton, OR) script and visualized with Prism 6 (GraphPad Inc., La Jolla, CA).

All these four points have been listed on a blacklist created by Jeffrey Beall – an academic librarian at the University of Colorado in Denver – which he published on his blog, Scholarly Open Access (https://scholarlyoa.com, cached on https://beallslist.weebly.com/) initially in 2010. This is known as the Beall's list.^13^ Because the companies producing these journals had the goal only to extract money from authors, he described them as ‘predatory journals/publishers’.^13^ Beall's list of criteria included five major points: editor and staff; business management; integrity; poor journal standards/practices and other. In the subpoints of this list, he extensively described what needed to be classified as unscientific practices and which also included the four findings of John Bohannon.^12^ In fact, Beall's list grew continuously over time and, although Beall offered all journals (Fig. 2a) and publishers (Fig. 2b) the option to appeal their listing, only a minority of these successfully appealed and were removed from the list. Although Beall's list has proved a useful tool for many scientists, Beall has been criticized for several reasons by many journals, publishers and scientists. Firstly, it has been argued that the methodology Beall used to classify a journal or a publisher as predatory was weak as it as mainly based on Beall's subjective impression and lacked transparency.^13^, ^14^ Secondly, some publishers complained that Beall did not contact them directly when issues about the editorial process or peer review process arose. Thirdly, critics said that Beall added newly started journals too quickly to his list. One of the challenges faced by editors of newly launched journals is their inherent lack of experience regarding the proper management of a scientific journal, which results in the creation of inferior websites. For this reason, these new journals match a multitude of criteria of Beall's list and are placed on the list before they are given the chance to improve.^13^, ^15^ Fourthly, Beall has been accused of having a general problem with the OA movement and therefore might not be the neutral party needed to create and maintain such a list.^14^ However, after being placed under heavy pressure from his employer – the University of Colorado – and receiving legal threats, Beall decided to remove his lists from his blog in January 2017.^16^, ^17^

Although Beall's list had certain shortcomings, it represented a valuable tool that researchers could use to assess journals on the basis of their credibility, raised awareness about this important issue and provided guidance for other institutions to create their own blacklists.^14^ Unlike Beall's blacklist, the Directory of Open Access Journals (DOAJ) has been designed as a whitelist, whereby it has defined a list of criteria (see Tables 2 and S2) that a journal has to fulfil to be included in the DOAJ (statistics on the DOAJ can be found in Fig. 2c). However, no list that is both updated and perfect is currently available, and there are still some ways to go around these lists. For this reason, several authors have urged publishing companies to establish standards that will allow them to discriminate predatory journals from serious, scientific publishers.^18^

**Table 2:** Criteria identified or suggested in the literature that can potentially be used to identify predatory journals

Criteria	Description	References
Peer review	Only superficial or no peer review process is provided by the journal to ensure scientific quality of the submitted paper	28
Emails	Aggressive or flattering email invitations sent to a large number of individuals to attract paper submissions from scientists	21
Advertising	Rapid publication/rapid peer review processes are promised, and low submission fees are advertised	32
Publication fees	Publication fees are hidden or only disclosed after the paper has been accepted	33
Title and logo	The journal's title can be misleading, mimic, or even cloning titles from well‐known prestigious journals, or can sound too ambitious. Also, the journal's logo can resemble that of a reputable journal	12, 35, 36
Editors	Fake (non‐existing) editors or the names of well‐known authors without their approval may be added to the editorial boards	31
Metrics	False impact factors or ‘fake metrics’ are provided to attract paper submissions	57
Contact information	No valid contact information (email, telephone number, address) is provided, and there is no possibility to get in touch with the publisher. Non‐professional email addresses from public providers (e.g. Yahoo, Gmail) are commonly used	37
Scope	The journal's scope is too broad, covering almost all fields of science	37
Publishing ethics and standards	Research and publishing ethics are not followed; reviewing, editing and or indexing services are not provided	16, 19
Indexing	Predatory publishers claim to have their articles indexed, while they are, in fact, not indexed in any important databases such as MEDLINE, PubMed and Web of Science	40
Copy‐editing and spelling errors	Published articles are poorly copy‐edited and contain numerous typographical or grammatical errors. In addition, such errors can be found on the journal's website, which also commonly include dead links	22
Submission system	Predatory journals ask authors to send their manuscripts by email, instead through a professional manuscript submission system	32

## Characteristics of predatory journals and impact on daily work

**Figure 3:**
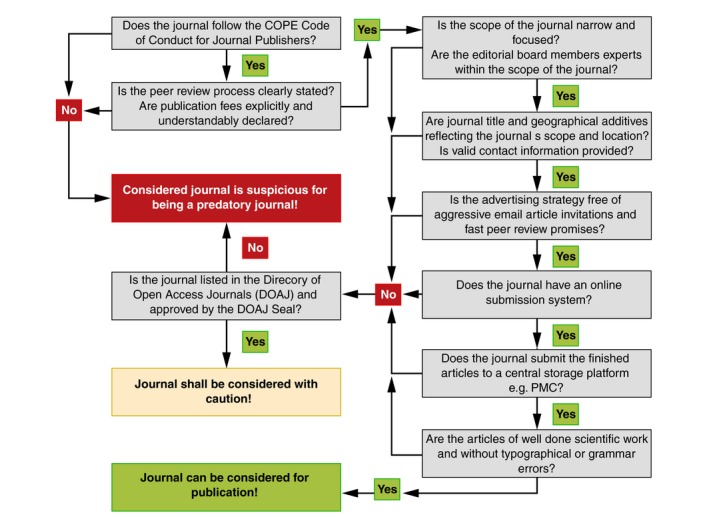
Decision tree that can be used by authors to discriminate between OA journals that are potentially suitable for article submission and predatory journals. COPE, Committee on Publication Ethics (https://publicationethics.org/).

Although several authors have suggested some criteria that can be used to identify predatory journals (summarized in Table 3 and shown in Fig. 3), it is still significantly challenging to differentiate among journals that have newly emerged and use questionable methods to attract paper submissions. Some of these companies are basically serious about establishing and operating a scientific journal that can be differentiated from ‘real’ predatory journals.^19^

**Table 3:** Criteria to receive the Seal of Approval for Open Access Journals (*DOAJ Seal*) by the Directory of Open Access Journals (DOAJ)

DOAJ Seal Criteria
The DOAJ Seal is given to journals that fulfil the following criteria: a permanent identifier within the published papers is provided provide DOAJ with article metadata deposit content with a long‐term digital preservation or archiving programme embed machine‐readable, CC licensing information in articles allow generous reuse and mixing of content, in accordance with a CC BY, CC BY‐SA or CC BY‐NC licences have a deposit policy registered with a deposit policy registry allow the author to maintain the copyright without restrictions
Available at https://doaj.org/publishers#seal; Accessed 20 November, 2017

Two major points on Beall's list were (i) the lack of rigorous control of scientific quality of the submitted manuscript due to the absence of or minimal nature of the peer review process and (ii) the efforts made by publishers to attract authors and encourage them to submit their manuscripts by aggressively emailing them, advertising rapid review processes and creating a false image of a reputable journal, providing falsified impact factors or fake editorial members.^19^, ^20^

Moher and Srivastava showed that a small number of publishers accounted for the majority of the invitations and that they could also be found on Beall's list.^21^ Characteristically, these emails begin with a flattering language and express praise for the author's recent work to encourage the submission of articles. They often contain numerous spelling and grammatical errors, which are indicative of their poor‐quality standards.^22^ More importantly, these emails sent out by illegitimate publishers are primarily sent to young, inexperienced researchers and especially those living in low‐ and middle‐income countries or working in growing disciplines, such as nursing. Not surprisingly, these authors are the main clientele of these predatory journals.^23^, ^24^ In consequence, researchers should simply ignore, junk or delete such emails.^25^

Companies that publish predatory journals are constantly searching for editorial board members and, if possible, scientists with high reputations,^2^ but do not run any background check on them. Sorokowski and colleagues sent 120 applications to journals listed in the Journal Citation Report (JCR; journals with an official impact factor), in the DOAJ, or on Beall's list. Forty journals on Beall's list, eight journals listed in the DOAJ and no journal listed in the JCR accepted the fake editors.^26^ However, one has to keep in mind that some journals, especially those that publish their articles in languages other than English, tend not to be listed in PubMed or the JCR. This is due to the fact that such articles are rarely cited, although they are fully peer reviewed and fulfil scientific standards.

Furthermore, companies publishing predatory journals often advertise their rapid peer review and fast‐track publication processes, citing that these take hours or days and rarely request any revisions, which is in direct opposition to the practices followed by prestigious journals.^27^, ^28^ The lack of rigorous peer review process is accompanied by a lack of the standards and good practices that have been established by the scientific community.^29^ However, the traditional peer review process, which adheres to these standards and good practices, is an essential tool that editors and reviewers use to screen and investigate scientific papers written by other researchers in the field and identify poorly executed papers or plagiarism. Unfortunately, editors of these journals are having difficulties finding adequate reviewers, as these are rarely paid, rewarded in any other way, and the burden of articles being published is continually increasing.^30^

In addition, researchers can recognize several other signs of journals with low scientific standards: for instance, the publisher/owner of the journal and the editor in chief may be listed as the same person, and the publisher might not be clearly identifiable, or may not have the required affiliations.^28^ The website may lack contact information (email, phone number and address), or the indicated information can be fraudulent.^31^ Legitimate publishers provide access to an accepted manuscript submission system in almost 100% of the cases, while predatory journals have been shown to request authors to send the articles by email in nearly 70% of the cases. Shamseer and colleagues could also show that most predatory journals were based in developing countries, possibly explaining why the true operating country of the publisher is frequently not stated on the website of the journal.^32^ In conclusion, authors should check whether the publisher is genuine and the contact details (especially address and phone number) are listed and valid, as these items seem to be good predictors of legitimate publishers.

Other fraudulent practices include predatory journals being dishonest about the APC or not clearly stating the publishing costs. In some cases, only after the article has been accepted are the authors charged with unreasonably high fees (up to $4000 if credit card information has already been given) or are the high submission fees disclosed, leaving the authors no other choice than to pay the money to get their articles published and giving them no chance to recover the money.^28^, ^33^ Once the work has been submitted, there is little chance that any attempts to retract the paper will be successful, as most of these bogus journals have a non‐cooperative retraction policy.^34^

Predatory journals often mimic titles or logos of prestigious, well‐known journals to confuse less experienced researchers.^35^, ^36^ The titles of such journals often sound quite ambitious and include words such as ‘Innovative’, ‘World’, ‘International’, ‘Global’, ‘American’, or ‘European’, covering almost all scientific areas.^37^ Another disturbing trend is the fact that cybercriminals register a domain name in order to create fraudulent websites for a counterfeit journal, which are designed to look identical to those of a legitimate scientific journal (‘hijacking’).^38^

Another important characteristic of predatory journals is their use of misleading metrics. Predatory journals frequently claim to have a current (Thomson‐Reuters) and high impact factor, or provide fabricated impact factors like the Journal Impact Factor (JIF), Universal Impact Factor (UIF), or Global Impact Factor (GIF), which are compiled by bogus companies. Sometimes, these counterfeited metrics are even made; researchers can check these using the Journal Citation Reports (JCR), and the metrics can be verified using Scopus.^39^ Indexing represents an important point for authors, and therefore, predatory publishers claim that they are indexed in major databases including MEDLINE, PubMed and Web of Science. In fact, predatory journals rarely provide sufficient indexing services, which results in the fact that researchers might not be able to search and find the article after it has been published. As these databases have different selection criteria, authors should not solely rely on a single database.^40^

## The problem of predatory journals for scientists

Predatory journals may put well‐established, scientific OA journals under pressure, as many authors might tend to publish their work in a predatory OA journal to avoid their article undergoing a full and time‐consuming peer review, as it would in a good OA journal.^41^

However, this raises the questions what the consequences of publishing in a predatory journal might be.

### Damaging external reputation

Increased attention should be paid as to where papers have been published.^25^, ^42^ A publication in a predatory journal might not be neutral on a CV and might even be an active demerit that harms the reputation of everyone, especially young scientists, listed on the article.^40^, ^41^

### Inexperience and lack of knowledge

Young researchers are inexperienced in the process of publishing and therefore unaware of predatory journals.^32^, ^43^ In this situation, companies publishing predatory journals offer the young scientists, who are often frustrated by a series of rejections, rapid peer review processes and publication times.^21^, ^32^, ^41^, ^44^, ^45^ Sometimes, the authors mistrusts the quality of their own work and would rather publish their work in a predatory journal.^41^

### Distribution

The potential advantage of not paying for access to articles in predatory journals seems attractive to most of us, but this can turn into a major disadvantage as the majority of laypersons might not be able to differentiate between data retrieved from a legitimate scientific journal and a predatory one. This might be particularly and seriously danger for clinicians if they consider the results published in such flawed articles in their decision‐making processes regarding patient treatment.

### Lack of quality control and reproducibility

The main purpose of the peer review process is to identify methodological or ethical weaknesses in a scientific paper.^11^, ^46^ The majority of errors found during the course of the peer review process are due to the lack of experience or knowledge.^47^ However, unscrupulous scientists may also take advantage of the lack of peer review process in predatory journals to publish flawed studies or questionable results.^16^, ^48^ Scientists even have successfully published flawed scientific papers about Star Wars and completely computer‐generated, scientific articles to highlight the fact that these journals have no quality control whatsoever.^49^, ^50^ Once published, this flawed content can reappear in other articles, cited as references even in an article in a legitimate scientific journal.^40^, ^51^

### Loss of information

As most OA journals are only distributed online (i.e. no print version of the journal), it is of great importance that the articles are maintained online even after the journal has been cancelled. Therefore, libraries and journals put enormous efforts into the digitalization of books and journals to keep them preserved for upcoming generations. However, most OA articles today are stored in the World Wide Web, and one important cost factor for a journal is its IT infrastructure. As predatory journals are only based on considerations of cost‐effectiveness, unprofitable journals may be closed and all published articles in that journal will be lost. Therefore, authors should be aware of this problem and only consider OA journals that store their accepted and published articles in public repositories, such as PMC.^25^, ^52^

### Concealed conflict of interests

Predatory journals also can be abused to hide potential conflict of interests:^53^ a very famous case – although not published in a predatory journal – was the case of Wakefield in the Lancet. This case demonstrates how one falsified study can continue to have tremendous effects on public health for decades. In his work, Wakefield linked the MMR vaccine with autism in children, which later was proven to be a false claim and led to the retraction of the article in 2004.^54^ However, the retracted articles till get continuously cited, although its claims have been proven wrong.

## Conclusions and outlook

Scientific journals and publishers play vital roles in the scientific community and, due to new publishing models such as OA, scientific journals and articles are now much more broadly distributed. In addition, more and more lay people can gain access to scientific literature due to the OA movement. This provides an important chance to increase the acceptance of science in the wider community but also represents a threat, because predatory journals can compromise this system. Therefore, journals and publishers should make efforts to strengthen this concept and contribute to more awareness of this topic. A new system has to be implemented to identify predatory journals and all articles published in such journals. As lay people and most doctors place their trust in the validity of scientific literature and cannot currently easily identify unscientific, non‐peer reviewed articles, this is a major issue, as these articles can destroy scientific trustworthiness and may influence physicians’ decisions in a harmful way. The JCR listing allows researchers to identify journals they can trust, but many doctors and especially patients are not aware of it. In cases of uncertainty, a scientifically experienced researcher should be consulted.

## Supporting information


**Table S1** PMC inclusion criteria
**Table S2** Inclusion criteria that a journal has to fulfil to be included in the Directory of Open Access Journal (DOAJ) list
