# Supplementary material for: Problems and challenges of predatory journals
Source: J Eur Acad Dermatol Venereol. 2018 May 29;32(9):1441–9. doi: 10.1111/jdv.15039 (PMC6174996; doi:10.1111/jdv.15039)
Supplement: Supplementary file 1 — Table S1 PMC inclusion criteria Table S2 Inclusion criteria that a journal has to fulfil to be included in the Directory of Open Access Journal (DOAJ) list [file JDV-32-1441-s001.docx]

**Supplements**

**Table S1.** PMC inclusion criteria*

| **PMC inclusion criteria** |
| --- |
| Scientific Quality Standard  US National Library of Medicine’s (NLM) Library Operations Division will decide whether the scientific and editorial character and quality of a journal merit its inclusion in PMC. In making this decision, NLM will consider the suitability of the journal for the NLM collection (based on the criteria in the **Collection Development Manual^#^** of the National Library of Medicine), as well as the opinions of expert consultants. Journals that already are fully indexed for MEDLINE generally will not require further scientific review for PMC. |
| Technical Requirements  A journal must provide PMC with the full text of articles in an XML (eXtensible Markup Language) format that conforms to an acceptable journal article DTD (Document Type Definition). PMC does not accept articles in HTML format.  NLM recommends that data be submitted in XML, conforming to the NISO JATS [Journal Publishing Tag Set](https://jats.nlm.nih.gov/publishing), but PMC will also accept data in other full-text article DTDs that are widely used in life sciences journal publishing.  Files required for each deposited article:   1. A separate XML data file for the full text of each article. 2. The original high-resolution digital image files for all figures in each article. 3. A PDF, if one exists, in addition to the XML version (but not as the only form.) 4. Supplementary data files (e.g., spreadsheets or video files) available with the article. |
| *PMC inclusion criteria are available in full detail at <https://www.ncbi.nlm.nih.gov/pmc/pub/addjournal/>, accessed 20 November, 2017  ^#^Available at <https://www.nlm.nih.gov/tsd/acquisitions/cdm/CDMBook.pdf>, accessed 30 December 2017 |

**Table S2.** Inclusion criteria that a journal has to fulfil to be included in the Directory of Open Access Journal (DOAJ) list.^#^

| **DOAJ Inclusion criteria** |
| --- |
| - The full text of ALL content must be available for free and be open access without delay (i.e., no embargo period); - The journal must have one URL for its website (one dedicated page to the journal only); - All the necessary journal business information pages (journal's aims and scope, the editorial board, the instructions for authors, the description of the quality control system, the open access statement, the plagiarism policy, and the licensing terms) must be hosted on this same site and not be held centrally on another website; - DOAJ also requests that a journal has its own dedicated website. No other service or product should be present under that URL. All the journal content that the publisher includes in the application must be in one place; - The content should be available article by article, with one unique URL for each article; - A journal must have at least an ISSN (International Standard Serial Number) that is registered at issn.org; - A journal's homepage should be clear, concise, and easy to navigate. It must contain visible links to the journal information pages. It must 'demonstrate that care has been taken to ensure high ethical and professional standards'; - A journal must have an editor and an editorial board. A site user must be able to find and contact the editor or members of the editorial board if needed. A link to the editorial board must be displayed prominently on the journal's homepage. The following minimal information must be made available on the editorial board page: the name and affiliation of the editorial board members; - The website must be up-to-date; - All articles must go through a quality control system (editorial or peer review) before publication and the exact type of review must be stated clearly on the website; - A link to detailed and comprehensive guidelines for authors (Instructions for Authors) must be clearly presented on the journal's homepage; - Every journal must display clearly on its website information about any charges for an author to process or publish a paper. This includes article processing charges (APCs), submission charges, page charges, colour charges, or any instance where money is required to complete the publication process. If a journal does not levy any charges, then this must be clearly stated; - The journal's open access policy must be clearly stated on the journal's website (not the publisher's own site). It should also be linked to from the homepage; - The full text of the articles of the journal should be freely available without embargo; - In all instances, the journal website must state clearly and precisely define the readers’ and authors’ terms of use and reuse when they submit an article or use the published content; |
| ^#^ Available in full detail at <https://doaj.org/publishers#advice>, accessed 20 November 2017 |
